# Exploring the Relationship between Mediterranean Diet Adherence and Subjective Well-Being among Greek and Cypriot Adults

**DOI:** 10.3390/nu16081238

**Published:** 2024-04-21

**Authors:** Georgia-Eirini Deligiannidou, Elena Philippou, Eirini Vasiari, Vanda Lopes de Andrade, Marika Massaro, Mihail Chervenkov, Teodora Ivanova, Rui Jorge, Dessislava Dimitrova, Tatjana Ruskovska, Lence Miloseva, Viktorija Maksimova, Katarina Smilkov, Darinka Gjorgieva Ackova, María-Teresa García-Conesa, Paula Pinto, Christos A. Kontogiorgis

**Affiliations:** 1 Laboratory of Hygiene and Environmental Protection, School of Medicine, Democritus University of Thrace, Dragana, 68100 Alexandroupolis, Greece; edeligia@med.duth.gr (G.-E.D.); vasiari.nutrition@gmail.com (E.V.); 2 Department of Life Sciences, School of Sciences, University of Nicosia, Nicosia 1700, Cyprus; 3 Department of Nutritional Sciences, King’s College London, London SE1 9NH, UK; 4 Agriculture School, Polytechnic University of Santarém, 2001-904 Santarem, Portugal; vanda.andrade@esa.ipsantarem.pt (V.L.d.A.); paula.pinto@esa.ipsantarem.pt (P.P.); 5 Research Centre for Natural Resources, Environment and Society (CERNAS), 3045-601 Coimbra, Portugal; 6 Life Quality Research Centre (CIEQV), 2040-413 Rio Maior, Portugal; 7 Research Institute for Medicines (iMed.ULisboa), Faculty of Pharmacy, Universidade de Lisboa, 1649-003 Lisboa, Portugal; 8 Institute of Clinical Physiology, National Research Council (CNR), 73100 Lecce, Italy; marika.massaro@cnr.it; 9 Faculty of Veterinary Medicine, University of Forestry, 1797 Sofia, Bulgaria; vdmchervenkov@abv.bg; 10 Slow Food in Bulgaria, 9 Pierre De Geytre St. bl. 3, 1113 Sofia, Bulgaria; dessidim3010@gmail.com; 11 Department of Plant and Fungal Diversity and Resources, Institute of Biodiversity and Ecosystem Research, Bulgarian Academy of Sciences, 1113 Sofia, Bulgaria; tai@bio.bas.bg; 12 School of Health Sciences, Polytechnic University of Leiria, 2411-901 Leiria, Portugal; rui.jorge@ipleiria.pt; 13 Center for Innovative Care and Health Technology (ciTechcare), Polytechnic of Leiria, 2411-901 Leiria, Portugal; 14 Sport Sciences School of Rio Maior, Polytechnic University of Santarém, 2040-413 Rio Maior, Portugal; 15 Faculty of Medical Sciences, Goce Delcev University, Stip, Str. Krste Misirkov, No. 10A, POB 201, 2000 Stip, North Macedonia; tatjana.ruskovska@ugd.edu.mk (T.R.); lence.miloseva@ugd.edu.mk (L.M.); viktorija.maksimova@ugd.edu.mk (V.M.); katarina.smilkov@ugd.edu.mk (K.S.); darinka.gorgieva@ugd.edu.mk (D.G.A.); 16 Research Group on Quality, Safety and Bioactivity of Plant Foods, Centro de Edafología y Biología Aplicada del Segura-Consejo Superior de Investigaciones Científicas (CEBAS-CSIC), Campus de Espinardo, P.O. Box 164, 30100 Murcia, Spain; mtconesa@cebas.csic.es

**Keywords:** subjective well-being, Mediterranean diet adherence, Greece, Cyprus, lifestyle, life satisfaction

## Abstract

Associations between subjective well-being (SWB) and dietary habits, employment status, and habitual activities are increasingly capturing the focus of researchers as well as policymakers worldwide. This study aimed to explore these associations in a sample of the population in Greece and Cyprus via an online survey. In total, 936 questionnaires (470: Cyprus, 466: Greece) were analyzed to study the associations between the Mediterranean Diet (MD) (using the 14-item MEDAS score, (14-MEDAS)), subjective well-being (SWB), and several socioeconomic factors. Key remarks of this survey highlight the positive impact of MD adherence on some well-being items. Namely, statistically significant differences were found on the following items: Satisfied with life (*p* < 0.001), Life worthwhile (*p* < 0.001), Feeling happy (*p* < 0.001), worried (*p* = 0.005), and depressed (*p* = 0.001), when comparing Low MD adherence (14-MEDAS < 5) to High MD adherence (14-MEDAS > 10). Other lifestyle habits such as spending time with friends and family, spending time in nature, and habitual physical activity were associated with aspects of SWB such as Life satisfaction, Life worthwhile, Feeling happy, and energetic. The findings support adherence to the MD, since it is associated with higher life satisfaction and self-reported happiness in this sample and should be considered when developing health policies on well-being.

## 1. Introduction

The traditional Mediterranean Diet (MD), recognized by UNESCO as an Intangible Cultural Heritage of Humanity [1,2,3], emphasizes plant-based foods and is characterized by high consumption of minimally processed food such as unrefined cereals, fruit, vegetables, legumes, potatoes, nuts, and seeds; moderate consumption of dairy products, eggs, poultry, and fish; and low consumption of red meat. Extra virgin olive oil is the principal source of fat in the MD pattern, while wine may be consumed in moderation with meals [4]. There is ample and strong evidence from both observational and intervention studies that the MD is associated with lower overall mortality and the prevention of several chronic diseases such as cardiovascular disease, type 2 diabetes, metabolic syndrome, some types of cancers, and neurodegenerative diseases, among others [4]. This dietary pattern is also in line with the diet proposed by the EAT-Lancet Commission for a healthy and environmentally sustainable diet [5].

Recent years have seen the introduction of the concept of well-being in health-related disciplines [6], which is affected by social, economic, and health factors including diet. Well-being is a broad concept, integrating objective social and environmental factors, such as the availability of social support and sanitation facilities, unemployment, income, and education, but also the individual’s subjective assessment of their well-being referred to as subjective well-being (SWB) [7,8,9]. SWB reflects an overall evaluation of the quality of a person’s life from the person’s perspective or, in simple terms, the extent to which a person believes or feels that his/her life is going well [8]. The Organization for Economic Co-operation and Development (OECD), reflecting on the Guidelines on Measuring Subjective Well-Being [10], specify that SWB indicators have two sub-dimensions: (i) the hedonic sub-dimension, and (ii) the eudemonic sub-dimension [9]. Although research in SWB acknowledges that it cannot and should not be equated with overall well-being, the subjective nature of the construct is what gives it its power. This is because the objective circumstances of different people are weighed differently depending on factors such as their life’s aims, values, and culture, which are presumably better captured in subjective compared to objective evaluations of well-being [6].

In the past few years, there has been emerging evidence that diet may affect SWB. As a result of its higher content in antioxidant and anti-inflammatory foods, as well as folate and other B vitamins and polyunsaturated fatty acids, the MD is associated with the prevention of both physical and mental disorders and thus, results in better overall health and possibly perceived well-being [11]. Indeed, in recent years, some observational studies have linked the MD with a higher quality of life [12,13]. In various cohorts of both older participants [14,15,16], younger participants [17], and mixed ages [18], adherence to the MD has been associated with a higher quality of life and/or life satisfaction [19]. In Australian adults, higher intakes of plant foods associated with an MD pattern are positively associated with physical function and general health and negatively associated with depression and perceived stress [20]. Moreover, in an Italian cohort, the association between adherence to the MD and better health-related quality of life is largely explained by higher associations of fruit, vegetable, and legume consumption with nearly all domains of quality of life, linking the outcome to a higher consumption of antioxidants and, at a lower extent, of dietary fiber, as whole grains were only partially associated with the evaluated domains [18].

Our group, which represents the Mediterranean Diet and Wellbeing (MeDiWeB) consortium, has recently documented, for the first time, a positive association between SWB and the 14-item Mediterranean Diet Adherence Screener (14-MEDAS) score in Portuguese adults, according to which, a higher adherence to MD (described as a higher 14-item MEDAS score) was observed in participants with higher SWB (including both eudemonic and hedonic items) [9,21].

On the other side of the coin, previously published systematic reviews and meta-analyses shows that lower adherence to MD, the tendency to move towards Western diet patterns, and a higher Dietary Inflammatory Index were associated with increased odds of incident depression [22,23,24]. As previous research reports, these associations with depression are hypothesized to be due to higher levels of inflammation [25,26]. Also, reflecting on particular food choices, previous research highlights that higher fruit and vegetable intakes are associated with a reduced risk of incident depression, while no association was observed between the Mediterranean diet, “healthy” diet, fish intake, and incident depression [24]. Since some studies have observed no associations between diet and depression or between adherence to the MD and psychological well-being, further research is necessary to delineate this relationship considering possible confounding or related factors such as education, socioeconomic factors, and lifestyle behaviors such as stress, sleep, social connections, and physical activity [27,28]. 

It is widely acknowledged that the last decades have seen a nutrition transition towards the Western diet characterized by increased consumption of processed foods and associated total, saturated, and trans fats and sugar [29,30]. In particular, a multicenter study assessing global data showed that countries in the Mediterranean basin, including Greece and Cyprus, are drifting away from the MD pattern [31]. Indeed, in a previous publication by our group, we have documented a moderate adherence to the MD in both countries [32]. Furthermore, it is worth noting that studies conducted after the COVID-19 pandemic have demonstrated key variances in the food choices of populations especially an increase in ultra-processed food consumption, which are of concern [33].

Although previous large-scale studies have evaluated the effect of MD on the health status and quality of life in the population of Greece [34,35] and Cyprus [36,37], to our knowledge, studies associating adherence to the MD with subjective well-being in Greece or Cyprus are scarce. Taking all of the above into consideration and since Greece and Cyprus share a similar diet and other cultural (e.g., religion, language) factors, we aim to assess any associations between subjective dimensions of well-being with MD adherence and lifestyle habits in a sample of the population in Greece and Cyprus.

## 2. Materials and Methods

### 2.1. Study Design

The current cross-sectional study is part of a research project carried out under the frame of MeDiWeB, the scope of which, as well as the English version of the questionnaire, has been fully described and published elsewhere [21].

This work describes the outcomes referring to data collected through a self-completed online questionnaire (MeDiWeB questionnaire) from a sample of participants in Greece (GR) and Cyprus (CY). In brief, the questionnaire was structured to evaluate and study the potential correlations of three main domains: (i) Adherence to the MD via the 14-item MEDAS score (14-MEDAS); (ii) Subjective well-being (SWB) via a total of 11 questions from previously validated instruments (including five questions as part of the spectrum of life satisfaction and anxiety perception: “Satisfied with life”, “Life worthwhile”, “Feeling happy”, “Feeling worried”, and “Feeling depressed”, three questions as part of the spectrum of feeling fatigue: “Feeling energetic”, “Feeling efficient”, and “Feeling tired” assessed via a Likert-type scale from 0 = not at all to 10 = completely, and three questions as part of the spectrum of emotional well-being: “Feeling nervous and stressed”, “Feeling unable to cope”, and “Feeling confident to handle problems” assessed via a Likert-type scale from 1 = not at all to 5 = completely); and (iii) Socioeconomic and Lifestyle factors, such as income, employment status, education level, etc. The questions were assessed either via a Likert-type scale or via multiple-choice options provided to the respondents.

### 2.2. Ethics

The questionnaire was approved by the Ethics Committee of each partner research institution (Ethics Committee of Research, Democritus University of Thrace, Approval Code: A. Π.: ΔΠΘ/ΕHΔΕ/3457/10, Approval Date: 16/09/2019 and Cyprus Bioethics Committee, Approval Code: EEBK EK 2019.01.100, Approval Date: 24/05/2019) and complies with the European Regulation on Data Protection. The questionnaire was provided online to the population of both countries in the Greek language following an assessment of the accuracy of the translation of the questionnaire in a pilot sample. Responses were confidential and anonymous. Potential participants were approached via mailing lists, online sharing in communication and networking media, and word-of-mouth. Participation in this survey was voluntary, and informed consent was requested from the participants before responding.

### 2.3. Data Collection

The questionnaire was constructed in Google Forms and disseminated as previously described. Data were collected between April 2019 and the end of the year 2020 (data evaluated in this survey are cut off until mid-March, i.e., before the COVID-19 lockdown) in the population of Cyprus (CY) and Greece (GR).

The data were collected via snowball sampling whereby, in each communication, the potential participant(s) were asked to forward the questionnaire to other interested parties. Participants who indicated (i) lack of consent, (ii) age < 18 years, (iii) duplicates, and/or (iv) participants whose nationality differed from the country in which they were living were eliminated from the study. A total of 936 adults (age ≥ 18 years) distributed across the two countries were finally included in the analysis.

### 2.4. Statistical Analysis

All variables were properly coded and qualified as nominal, ordinal, and scale variables. Scale variables are presented as median (IQR, interquartile range) and mean ± SD. Frequencies and percentages are used for ordinal or nominal variables. The normality of the distribution of data has also been evaluated (Shapiro–Wilk test). Non-parametric tests such as Mann–Whitney U test and Kruskal–Wallis one-way ANOVA were used when normality was not followed, and ANOVA tests were used to assess differences between the variables for normally distributed variables, respectively. (All statistical tests were based on a significance level of 5% (α = 0.05). Statistical analyses were performed using the Statistical Package for the Social Sciences (SPSS) statistical package for Windows (SPSS, Inc., Chicago, IL, USA).

## 3. Results

### 3.1. Data Evaluation and Screening

A total of 965 responses were initially obtained at the time of the online survey cut-off. All responses were screened, and 29 were excluded from further analysis, as they did not comply with the inclusion criteria (13: lack of consent, 2: age < 18, and 14: described nationality other than GR or CY). Overall, 936 responses were included in the analysis presented in the results. Missing data in some variables (such as education level, marital status, etc.) may show as differences in the number of total responses.

The normality of the distribution of data (age, BMI, income, MEDAS-14 score, and SWB items), evaluated via the Shapiro–Wilk test, is available in Supplementary (Figure S2, Table S3). Non-parametric tests were used when the normal distribution of data was not followed (Mann–Whitney U test or the Kruskal–Wallis one-way ANOVA). Given the data collection method for this survey as previously presented, the representability of our sample leads us to highlight that the findings ought not to be generalized for either of the populations. However, the associations of MD adherence to SWB remain relevant and will be addressed further in the results.

### 3.2. Population Demographics and Comparison between Countries

Table 1 describes the overall demographics of the population studied. In brief, the majority of responders in both countries were female (GR: 76.8% and CY: 71.6%), with a mean age of 35.1 ± 9.8 and 38.7 ± 12.1 years old for Greece and Cyprus, respectively.

Mean and median income for each country were grouped into three classes (low, medium, and high) based on the 2019 and 2020 average values of mean income available by Eurostat [38]. The average monthly personal income was calculated based on these values. The mean personal income was 1.836 EUR/month and 3.721 EUR/month, for respondents from Greece (average monthly calculated income: 707.3 EUR) and Cyprus (average monthly calculated income: 1.371.6 EUR), respectively. Additionally, more than half of the responders in both countries had higher education (university/master’s degrees) and were married or in a relationship, while the majority (GR: 274 (58.80%) and CY: 388 (82.70%)) were employed at the time of this survey. 

Regarding the key health-related risk factors of smoking and being overweight, an average of 23% of respondents for both countries were reported as current smokers, and over 40% had Body Mass Index (BMI) values (based on self-reported weight and height) corresponding to the overweight or obese classifications according to the calculated index. Overall, 75.00% of respondents had an MD adherence score corresponding to the medium classification (score from 6 to 9 points), and it is worth noting that the maximum score (14-MEDAS = 14) was not reached by any of the responders in either country. Additional records of the distribution of our sample population in other lifestyle factors are available in Table S1 (Supplementary).

### 3.3. SWB Score vs. 14-MEDAS Categories

As an initial step to evaluate the 14-MEDAS impact on the participants’ SWB, 14-MEDAS scores were classified as low, medium, and high as previously described [9]. The mean values of the items evaluating SWB were calculated for each 14-MEDAS class (Table 2) and tested for homogeneity of variances (Levene statistic). The same was performed for BMI. Since the homogeneity was not true for all items, the Kruskal–Wallis one-way ANOVA was carried out to identify differences between the 14-MEDAS groups concerning the BMI values and the SWB items.

As shown in Table 2, 14-MEDAS scores were associated with most of the SWB items in a statistically significant way, while the association with BMI was borderline significant (*p* = 0.047). Although this is a cross-sectional study and, thus, causality cannot be inferred, a positive association was demonstrated between higher 14-MEDAS scores and more positive responses to the majority of the evaluated SWB items (Figure S1). It is worth noting that low and medium 14-MEDAS scorings had little effect on the response means of the SWB items, whereas high 14-MEDAS scoring was associated with notable changes in the response means of the SWB items. This was particularly evident in the evaluations on feelings of worry, tiredness, and depression. Additionally, medium and high 14-MEDAS classes were associated with lower (within normal range) BMI values.

As a following step, a Principal Component Analysis of the 11 items of SWB was conducted to evaluate the top five more relevant factors by 14-MEDAS class and sex. The Kaiser–Meyer–Olkin Measure of Sampling Adequacy was 0.782 (*p* < 0.001), thus reflecting the adequacy of our data for this analysis. It was shown that the items “Satisfied with life”, “Life worthwhile”, “Feeling happy”, and “Feeling worried” were the primary factors (with Eigenvalues λ > 1) able to describe up to 72.01% of the total sample. These factors were followed by feeling depressed (λ = 0.782), energetic (λ = 0.631), and efficient (λ = 0.434), which could collectively describe up to 88.8% of the sample. The description of these factors is presented in Table S2 (Supplementary). Seeing that the mean values of the male and female participants were similar in the PCA analysis, the Kruskal–Wallis H test was used for the comparison of SWB items (regardless of 14-MEDAS classification) between male and female participants, showing that the items “Feeling happy” (*p* = 0.017), “Feeling worried” (*p* < 0.001), and “Feeling depressed” (*p* < 0.001) were statistically different between sexes.

Differences between the 14-MEDAS_Groups of Low, Medium, and High on the distinct SWB items were also tested via the independent samples Kruskal–Wallis test. Statistically significant differences between the 14-MEDAS_Groups were found on most of the SWB items: “Satisfied with life” (*p* < 0.001), “Life worthwhile” (*p* < 0.001), “Feeling happy” (*p* < 0.001), “Feeling worried” (*p* = 0.005), “Feeling depressed” (*p* = 0.001), “Feeling energetic” (*p* = 0.001), “Feeling efficient” (*p* = 0.001), and “Feeling tired” (*p* < 0.001). No significant differences were found for the SWB items: “Feeling nervous and stressed” (*p* = 0.342), “Feeling unable to cope” (*p* = 0.450), and “Feeling confident to handle problems” (*p* = 0.337), in which cases, there was no significant evidence of differences across groups. The same test evaluated where the differences lie by comparing the 14-MEDAS_Groups in pairs (i.e., Low vs. Medium, Medium vs. High, and then, Low vs. High) for each of the distinct SWB items. In this case, differences were rendered significant in the comparisons against the 14-MEDAS_Group High for all SWB items and for 14-MEDAS_Group Low vs. 14-MEDAS_Group Medium for “Satisfied with life” (*p* = 0.027), and “Life worthwhile” (*p* = 0.003). The results of this analysis were further evaluated for effect size for the statistically significant differences, resulting, in all cases, in only weak associations.

Potential correlations between the SWB items and 14-MEDAS classes were investigated using Spearman’s rho correlation coefficient. As previously reported, income was significantly correlated with the item “Feeling nervous and stressed” at a low rate of 0.248 in the evaluation of the sum of the population studied, while in the evaluation of the participants grouped by country, several more correlations appeared to also have some small correlations. Namely, in participants from Cyprus, statistically significant (*p* < 0.01) positive correlations were rendered for SWB and the items “Satisfied with life” (0.261), “Life worthwhile” (0.212), “Feeling happy” (0.219), and “Feeling efficient” (0.214), and a negative correlation was reported for “Feeling depressed” (−0.206), while the same items appeared in the analysis of participants from Greece with smaller correlation values.

Furthermore, all SWB items were statistically significantly correlated with each other, with the highest correlations observed between “Satisfied with life” vs. “Life worthwhile” at 0.687 (*p* < 0.001) and vs. “Feeling happy” at 0.650 (*p* < 0.001), as well as “Feeling energetic” vs. “Feeling efficient” at 0.775 (*p* < 0.001). Additionally, the 14-MEDAS score (evaluated as a single variable without the classification) was correlated with “Life worthwhile” (0.212, *p* < 0.01), and “Sports frequency” was correlated with “Feeling energetic” (0.259, *p* < 0.01).

Additionally, “Sex life satisfaction” was also evaluated as a separate item of lifestyle factors contributing to the SWB of the participants. This item was correlated with “Satisfied with life”, “Life worthwhile”, and “Feeling happy” (*p* < 0.01), although the correlations were relatively low (0.374, 0.343, and 0.391, respectively). The same item was also correlated with “Feeling depressed” (0.300), “Feeling energetic” (0.233), and “Feeling efficient” (0.256), although the correlation coefficients were even lower but still statistically significant (*p* < 0.01). Interestingly, statistically significant correlations (*p* < 0.01) between nationality and the SWB items “Feeling nervous and stressed” (0.538) and “Feeling confident to handle problems” (0.499) were also observed.

The results led to further investigation of the potential effects of other lifestyle factors on the more characteristic SWB items for our population sample as previously described. In this setting, Table 3 describes the outcomes of the pairwise comparisons (using the Kruskal–Wallis H test) between some lifestyle factors (smoking, spending time in nature, spending time with family, spending time with friends, and habitual activity type) and the SWB items. Results are presented in mean (SD) values, and the significance of the correlation is described by the subscripts.

Smoking status seemed to be affecting most of the SWB items evaluated except “Feeling depressed”, and “Feeling tired”. Also, “Spending time with family” was positively correlated with the SWB item “Satisfied with life” (0.207, *p* < 0.01) when the participants from Greece and Cyprus were evaluated separately. Additionally, “Habitual activity type”, as well as “Sports frequency”, were positively correlated (*p* < 0.01) with “Feeling energetic” (0.261 and 0.259, respectively) and “Feeling efficient” (0.247 and 0.224, respectively), and the activity type was also negatively correlated with “Feeling stressed and nervous” (−0.207, *p* < 0.01).

## 4. Discussion

### 4.1. SWB and MD Adherence Co-Assessment

In a sample of adults from two Mediterranean countries, namely, Greece and Cyprus, with similar diets and other cultural characteristics, we have shown for the first time that higher adherence to the MD and other lifestyle factors are associated with higher subjective well-being. In particular, we found significant associations between higher adherence to the MD and increasing positive aspects of SWB such as “Feeling happy”, “Satisfied with life”, and “Life worthwhile” and with lower negative aspects such as “Feeling worried” and “Feeling depressed”. In addition to the MD, we have also shown that other lifestyle habits, such as spending time with friends and family, spending time in nature, habitual physical activities, and not smoking, are all associated with a higher SWB.

The results are in agreement with the previous findings of our group—the MeDiWeb consortium—in studies in the Portuguese population [9] and in Mediterranean and non- Mediterranean countries [39]. Previous research has also shown that a healthy diet including adequate amounts of food groups such as fruit, vegetables, and low sweet consumption is associated with well-being [40,41]. It is worth noting that studies conducted during the COVID-19 pandemic and/or lockdown, reflecting its impact on the lifestyle in populations of both Greece and Cyprus, have demonstrated that, during that time, there was an increase in the quality of dietary choices [40,41]. Since then, however, there has been a concern regarding the general increase in consumption of ultra-processed foods [33]. Thus, although the association of diet with subjective well-being remains valid, the outcomes of this study regarding MD adherence and SWB, separately, are to be examined as a reflection of our sample for the study period and, therefore, cannot be generalized. Nevertheless, the associations are complex due to cultural, religious, socioeconomic, and possibly residual confounders making it difficult to delineate the direction of this relationship; thus, further investigation is necessary [39].

It is of note that as previously published by our consortium [32], adherence to the MD in these two Mediterranean countries is only moderate, a disappointing finding, considering their deep roots in the Mediterranean culture, as well as the overall high educational level of our sample, which, in fact, probably biased the results towards a higher MD adherence. This finding shows that the dietary recommendations are being overlooked as both countries move towards a Westernized nutrition transition. All the above findings should be taken into consideration when trying to develop policies on diet and health.

### 4.2. Factors Associated with SWB: Exploring the Relationship with Sociodemographic and Lifestyle Factors

Our findings provide further support that lifestyle habits such as spending time with friends and family, spending time in nature, habitual physical activity, and not smoking are associated with SWB and specifically positive aspects such as life satisfaction, life worthwhile, feeling happy, and feeling energetic. These findings are also in agreement with our previous work including 2400 participants from Spain, Italy, Portugal, Bulgaria, and the Republic of North Macedonia where, using a 9-item SWB score, we confirmed that several Mediterranean lifestyle characteristics, such as higher adherence to the MD, adequate rest (sleeping longer at night), being active (higher daily normal activity, leisure activity, and sport practicing), spending more time socializing with family and friends, and spending more time in nature and not-smoking act as positive predictors of the SWB score. Sociodemographic and health-related variables such as older age, being a man, being married or being in a relationship, being employed, and not having a pathology also positively contribute to a higher SWB score. Overall, the combination of these variables explains around 17% of the variance in the SWB, thus indicating that further research is needed to determine which other variables might determine the remaining association [39] A study of the Spanish population has also shown that healthy lifestyle habits such as eating specific foods (fruits and vegetables), being in the company of family and friends, engaging in more exercise, and feeling healthy all had a positive influence on life satisfaction and happiness [42].

With regard to lifestyle factors and SWB, physical activity has been shown in a systematic review of observational and randomized controlled trials to be associated with happiness, although since the studies were observational, a causal association cannot be assumed [43]. It has also been shown that the emotional outlook of life predicts increases in physical activity among men who were initially inactive [44]. Furthermore, physical activity and well-being also contribute to better eating habits, especially in younger populations [17,45], which is important later in life. Conversely, low income adds to lower MD adherence, as there is a rise in dietary costs with growing prices of fruits and vegetables [46]. 

Strong social interactions, another one of the six pillars of lifestyle medicine [47], are associated with better mental and physical health. Indeed, we have shown positive associations between these factors and all the assessed SWB items except feeling energetic. Previous work in the Spanish population has shown positive associations between sharing meals with others and higher life satisfaction and happiness [42]. As reviewed by Diener [8], social relationships are consistently associated with a higher evaluation of one’s life satisfaction, with marital status being shown in systematic reviews to increase (albeit to a small extent) life satisfaction. This association, however, is still complicated by the fact that there seems to be adaptation to marriage, and associations are affected by culture and may change over time [8]. Additionally, even if SWB determinants were to be easily identified, current empirical research is still in search of a methodological protocol that can be unanimously used [48]. A strong network of close social relationships is also associated with higher levels of SWB, but these associations are small, possibly since relationship quality and quantity are not captured [49]. Finally, it is possible that personality characteristics leading to a broadly positive outlook on life further complicate these findings [8].

The present study has also replicated the findings of our previous publication in Mediterranean (Spain, Italy, Portugal) and non-Mediterranean countries (Bulgaria and Republic of North Macedonia), showing that women have a lower SWB perception than men [39]. This is also in agreement with data from 29 countries from the European Social Survey [50], thus confirming women’s higher likelihood to experience low/depressive symptoms. Again, more research is necessary both to better understand the factors associated with this finding, such as health, hormonal, socioeconomic, cultural, or other, and to design and develop more appropriate strategies or policies aiming to improve women’s SWB. In general, this study agrees with previous findings; nevertheless, it seems that overall, the associations between diet and other lifestyle factors with SWB are still limited to specific populations, interact between them and with SWB in a complex way, and, most importantly, do not explain fully the association between lifestyle and SWB suggesting that further research is necessary. 

### 4.3. Strengths, Limitations, and Future Research

This study has several strengths and limitations. With regard to strengths, it examined for the first time associations between the Mediterranean diet and lifestyle factors in a population sample of almost 1000 participants in Greece and Cyprus. Given the sample size as well as the methodology used to recruit participants, it must be noted that the outcomes of this research cannot be generalized. Compared to the national statistics of both countries, our sample was younger, more educated, more likely to be employed, and had a higher representation of women than men. Specifically, during the year 2020, over 38% of the population in Cyprus was of high education (had acquired a bachelor’s degree and have moved on to attending or had completed a master’s degree) [51]. Similarly, according to the OECD report, in Greece, 51% of 25–34-year-old women and 37% of their male peers had a tertiary qualification in 2020 [52]. Additionally, according to Elstat and ILO (International Labor Organization), approximately 7% of the population in Cyprus and 16% of the population in Greece were unemployed at the beginning of that year [53,54,55]. Furthermore, the male-to-female ratio of the citizens in both countries was approximately 1:1. Most of the responders in the present study, however, were female which may be related to the fact that women are more likely to self-select to participate in online surveys [56]. The other differences observed can be related to the study design, i.e., it being an online survey, but also to the snowball method of recruitment.

Additionally, although the study period was placed before the COVID-19 pandemic, since then, several changes have taken place concerning both what drives food choices as well as well-being in both populations. As mentioned above, both countries experienced a higher adherence to the MD during the COVID-19 lockdown and several lifestyle changes [57,58], but since then, there has been a general concern regarding the deterioration of food patterns across the world with the increasing use of ultra-processed foods [33]. Nevertheless, the associations relating MD adherence with the items of SWB remain relevant, as similar findings have been reported in larger samples of populations as previously discussed. Given the impact of dietary choices documented by this and other studies before, during, and after the pandemic on the physical and mental health of the populations, it is important to highlight the significance of steps taken to raise awareness and promote adherence to MD, such as the “Med-Index” [59]. The study also assessed various sociodemographic and health characteristics and different aspects of subjective well-being. Importantly, the SWB items used assessed both the hedonic and the eudemonic indicators of well-being thus providing a broader assessment of subjective well-being. To minimize the effect of possible bias due to cultural or language issues, only native citizens of the two countries were included in the analysis, and participants from other nationalities were excluded. The provision of an online questionnaire was both a strength and a limitation of the study since although it was easily accessible to the participants, it attracted a more middle-class, highly educated sample with possibly overall better health status, lifestyle, and well-being indicators. As we have previously reported in our study of five different countries, the age, education, and job status of our sample were more representative of the working-age adult population in Europe [32]. Finally, due to the cross-sectional nature of our study but also other similar studies, cause-and-effect associations cannot be established. Lastly, the complexity of the associations and interactions remains to be investigated.

## 5. Conclusions

In an era of nutrition and lifestyle transition, the growing relevance and need to both increase SWB and identify the multiple factors shaping well-being, as well as consider them in designing strategies and policies aiming to increase society’s flourishing, are of paramount importance. In a Mediterranean population, we have shown that both adherence to the MD and other lifestyle aspects such as spending time with friends and family and in nature, habitual exercise, and not smoking are associated with a higher SWB. Concerning research conducted during and after the COVID-19 pandemic, which is established as a factor that affected both the dietary choices of the population as well as their well-being, the outcomes of our work—although preceding—highlight a very relevant link between MD as a dietary pattern as well as a lifestyle pattern and SWB in items of both eudemonic and hedonic dimensions. Since life satisfaction and the self-reported happiness of individuals are increasingly acknowledged as playing important roles in health outcomes, it is important to consider these findings when developing public health policies aiming to improve physical and mental health. Future research should aim to identify other covariates that play important roles in well-being and effective interventions to improve the factors shaping well-being.

## Figures and Tables

**Table 1:** General population characteristics.

		GR	CY	Total
**Sex**	Male	108 (23.20%)	133 (28.40%)	241 (25.83%)
	Female	357 (76.80%)	335 (71.60%)	692 (74.17%)
Age * (years) Mean ± SD: 36.8 ± 11.2 Median: 35 Range: 18–75	18–29	152 (32.80%)	119 (25.30%)	271 (29.05%)
	30–39	173 (37.40%)	154 (32.80%)	327 (35.05%)
	40–49	96 (20.70%)	114 (24.30%)	210 (22.51%)
	50–59	31 (6.70%)	49 (10.40%)	80 (8.57%)
	60–69	11 (2.40%)	26 (5.50%)	37 (3.97%)
	>70	0 (0.00%)	8 (1.70%)	8 (0.86%)
**Education ^#^**	Primary Education	2 (0.40%)	1 (0.20%)	3 (0.32%)
	Secondary Education	52 (11.20%)	34 (7.20%)	86 (9.19%)
	University	243 (52.10%)	148 (31.50%)	391 (41.77%)
	Master/Ph.D.	169 (36.30%)	287 (61.10%)	456 (48.72%)
**Marital Status**	Single	161 (34.50%)	147 (31.30%)	308 (32.91%)
	Married/In a relationship	275 (59.00%)	305 (64.90%)	580 (61.97%)
	Divorced	29 (6.20%)	17 (3.60%)	46 (4.91%)
	Widowed	1 (0.20%)	1 (0.20%)	2 (0.21%)
BMI * (kg/m^2^) Mean ± SD: 25.11 ± 4.94 Median: 24.15 Range: 16–49	Underweight (<18.5)	13 (2.80%)	20 (4.30%)	33 (3.53%)
	Normal (18.5–25.0)	236 (51.10%)	268 (57.30%)	504 (53.85%)
	Overweight (25.1–30.0)	136 (29.40%)	113 (24.10%)	249 (26.60%)
	Obese (30.1–35.0)	48 (10.40%)	51 (10.90%)	99 (10.58%)
	Overly Obese_1 (35.1–40.0)	22 (4.80%)	10 (2.10%)	32 (3.42%)
	Overly Obese_2 (>40.1)	7 (1.50%)	6 (1.30%)	13 (1.39%)
**Employment status**	Student	81 (17.40%)	51 (10.90%)	132 (14.12%)
	Employed	274 (58.80%)	388 (82.70%)	662 (70.80%)
	Seeking the first job	14 (3.00%)	0 (0.00%)	14 (1.50%)
	Unemployed for part of the year	34 (7.30%)	16 (3.40%)	50 (5.35%)
	Unemployed	56 (12.00%)	0 (0.00%)	56 (5.99%)
	Pensioner	7 (1.50%)	14 (3.00%)	21 (2.25%)
Net income * (euros) Mean ± SD: 2800 ± 5882 Median: 1700 Range: 0–70000	Low	50 (12.60%)	80 (19.30%)	130 (16.03%)
	Medium	53 (13.40%)	54 (13.00%)	107 (13.19%)
	High	293 (74.00%)	281 (67.70%)	574 (70.78%)
**Smoking**	No	347 (74.60%)	369 (78.70%)	716 (76.66%)
	Yes	118 (25.40%)	100 (21.30%)	218 (23.34%)
14-MEDAS * Mean ± SD: 6 ± 2 Median: 6 Range: 1–12	Low (<5)	89 (19.10%)	101 (21.50%)	190 (20.30%)
	Medium (6–9)	355 (76.20%)	347 (73.80%)	702 (75.00%)
	High (>10)	22 (4.70%)	22 (4.70%)	44 (4.70%)

* For these variables, a Mann–Whitney U test was conducted to evaluate the differences between countries: Age (*p* < 0.001), BMI (*p* = 0.007), 14-MEDAS (*p* > 0.05), Net family income (*p* < 0.001). ^#^ Regarding the classes of education visit: https://eurydice.eacea.ec.europa.eu/national-education-systems, accessed on 30 March 2024. The class of Master/Ph.D. reflects the studies attended after obtaining a Bachelor’s degree (4–6 years depending on the major).

**Table 2:** Associations between BMI and SWB items by 14-MEDAS categories (Low–Medium–High).

	14-MEDAS_Groups ^+^						
	Low (<5)		Medium (6–9)		High (>10)		
	Mean ± SD	Median (IQR)	Mean ± SD	Median (IQR)	Mean ± SD	Median (IQR)	*p*-Value *
BMI (kg/m^2^)	25.75 ± 5.23	24.84 (6.36)	25.01 ± 4.92	24.03 (6.07)	23.90 ± 3.62	23.18 (6.13)	**0.047**
Satisfied with life ^+^	6.89 ± 2.00	7 (3.00)	7.21 ± 1.77	8 (2.00)	8.22 ± 1.18	8 (1.00)	**<0.001**
Life worthwhile ^+^	7.22 ± 1.81	7 (2.00)	7.66 ± 1.66	8 (2.00)	8.77 ± 0.83	9 (1.00)	**<0.001**
Feeling happy ^+^	6.58 ± 2.16	7 (2.00)	6.83 ± 1.96	7 (2.00)	8.04 ± 1.28	8 (2.00)	**<0.001**
Feeling worried ^+^	5.64 ± 2.63	6 (5.00)	5.54 ± 2.56	6 (5.00)	4.15 ± 2.73	4 (4.00)	**0.005**
Feeling depressed ^+^	4.01 ± 2.88	4 (6.00)	3.71 ± 2.77	3 (5.00)	2.20 ± 2.12	2 (2.50)	**0.001**
Feeling energetic ^+^	6.03 ± 2.20	6 (3.00)	6.34 ± 1.92	6 (3.00)	7.29 ± 1.77	7 (1.50)	**0.001**
Feeling efficient ^+^	6.37 ± 2.00	7 (3.00)	6.51 ± 1.86	7 (3.00)	7.47 ± 1.66	8 (1.50)	**0.001**
Feeling tired ^+^	5.82 ± 2.46	6 (4.00)	5.66 ± 2.42	6 (4.00)	4.04 ± 2.68	4.5 (4.00)	**<0.001**
Feeling nervous and stressed ^#^	2.85 ± 0.90	3 (1.00)	2.75 ± 0.88	3 (1.00)	2.75 ± 0.65	3 (1.00)	0.342
Feeling unable to cope ^#^	3.10 ± 1.05	3 (2.00)	3.15 ± 1.04	3 (2.00)	3.35 ± 1.16	3 (2.00)	0.450
Feeling confident to handle problems ^#^	2.53 ± 1.01	2 (1.00)	2.39 ± 0.97	2 (1.00)	2.34 ± 0.83	2 (1.00)	0.337
Employment satisfaction ^+^	7.03 ± 2.02	7 (2.00)	7.25 ± 2.02	8 (3.00)	7.73 ± 1.98	8 (2.00)	0.069

* Differences between 14-MEDAS groups were assessed using Kruskal–Wallis one-way ANOVA; *p* < 0.05 was considered statistically significant. ^+^ MEDAS scale from 0 (no adherence) to 14 points (total adherence); SWB items scale and Employment satisfaction scale from 0 (not at all/never) to 10 (completely/always). ^#^ SWB items scale from 1 (not at all/never) to 5 (completely/always).

**Table 3:** Associations of SWB items with lifestyle factors.

	Satisfied with Life	Life Worthwhile	Feeling Happy	Feeling Worried	Feeling Depressed	Feeling Energetic	Feeling Efficient	Feeling Tired
**Smoking**								
No	7.35 ± 1.74 _a_	7.77 ± 1.62 _a_	7.01 ± 1.95 _a_	5.39 ± 2.64 _a_	3.61 ± 2.79 _a_	6.42 ± 1.99 _a_	6.61 ± 1.89 _a_	5.56 ± 2.47 _a_
Yes	6.69 ± 1.96 _b_	7.14 ± 1.80 _b_	6.27 ± 2.04 _b_	5.86 ± 2.42 _b_	4.00 ± 2.74 _a_	6.04 ± 1.96 _b_	6.31 ± 1.88 _b_	5.80 ± 2.45 _a_
**Spending time in nature**								
Never	6.81 ± 2.02 _a_	7.26 ± 1.95 _a_	6.45 ± 2.35 _a_	5.92 ± 2.77 _a_	4.05 ± 3.00 _a_	6.25 ± 2.07 _a,b_	6.31 ± 2.10 _a_	5.90 ± 2.47 _a_
Occasionally	7.17 ± 1.68 _a,b_	7.58 ± 1.53 _a,b_	6.71 ± 1.80 _a,b_	5.28 ± 2.45 _a_	3.76 ± 2.66 _a_	6.17 ± 1.87 _a_	6.45 ± 1.76 _a,b_	5.47 ± 2.40 _a_
Sometimes	7.34 ± 1.71 _b,d_	7.77 ± 1.65 _b_	7.02 ± 1.83 _b,c_	5.41 ± 2.49 _a_	3.53 ± 2.70 _a_	6.36 ± 1.95 _a,b_	6.66 ± 1.77 _a,b_	5.52 ± 2.47 _a_
Frequently	7.47 ± 2.00 _b,c,d_	7.93 ± 1.73 _b,c_	7.41 ± 2.10 _c_	5.50 ± 2.81 _a_	3.34 ± 2.89 _a_	6.80 ± 2.17 _b_	6.92 ± 2.02 _b_	5.62 ± 2.65 _a_
Almost all the time	8.25 ± 1.42 _d_	8.25 ± 1.26 _a,b_	7.25 ± 2.01 _a,b,c_	5.75 ± 2.95 _a_	3.13 ± 2.89 _a_	6.42 ± 1.95 _a,b_	6.79 ± 1.82 _a,b_	6.00 ± 2.34 _a_
**Spending time with family**								
Never	6.49 ± 2.23 _a,b_	7.11 ± 1.95 _a,b_	5.49 ± 2.76 _a_	6.49 ± 2.76 _a_	4.83 ± 3.29 _a_	5.86 ± 2.06 _a_	6.09 ± 1.93 _a,b_	5.97 ± 2.38 _a_
Occasionally	6.66 ± 1.89 _a_	7.04 ± 1.91 _a_	6.33 ± 2.14 _a,b_	5.32 ± 2.22 _a,b_	3.88 ± 2.70 _a_	5.84 ± 2.02 _a_	5.68 ± 2.14 _a_	5.80 ± 2.51 _a_
Sometimes	6.95 ± 1.77 _a,b_	7.40 ± 1.91 _a,b_	6.73 ± 1.99 _b,c_	5.50 ± 2.50 _a,b_	4.06 ± 2.80 _a_	6.40 ± 1.82 _a_	6.63 ± 1.77 _b_	5.66 ± 2.41 _a_
Frequently	7.31 ± 1.75 _b,c_	7.65 ± 1.55 _b,c_	7.04 ± 1.85 _c_	5.18 ± 2.67 _b_	3.51 ± 2.70 _a_	6.39 ± 1.95 _a_	6.69 ± 1.83 _b,c_	5.49 ± 2.42 _a_
Almost all the time	7.51 ± 1.80 _c_	8.01 ± 1.51 _c_	7.04 ± 1.95 _c,d_	5.86 ± 2.62 _a_	3.52 ± 2.84 _a_	6.47 ± 2.07 _a_	6.67 ± 1.86 _b,d_	5.63 ± 2.56 _a_
**Spending time with friends**								
Never	5.91 ± 2.37 _a_	6.71 ± 1.86 _a_	5.42 ± 2.51 _a_	6.76 ± 2.53 _a_	5.09 ± 2.92 _a_	5.84 ± 2.33 _a_	5.69 ± 2.28 _a_	6.67 ± 2.33 _a_
Occasionally	7.11 ± 1.73 _b,c_	7.54 ± 1.60 _b_	6.64 ± 1.95 _b_	5.44 ± 2.55 _b_	3.64 ± 2.75 _b_	6.27 ± 1.83 _a_	6.42 ± 1.77 _a,b_	5.44 ± 2.52 _b_
Sometimes	7.08 ± 1.75 _b_	7.59 ± 1.73 _b_	6.81 ± 1.96 _b,c_	5.43 ± 2.52 _b_	3.86 ± 2.85 _b_	6.25 ± 2.05 _a_	6.61 ± 1.86 _b_	5.66 ± 2.39 _a,b_
Frequently	7.54 ± 1.68 _c_	7.89 ± 1.61 _b_	7.20 ± 1.83 _c_	5.43 ± 2.62 _b_	3.47 ± 2.75 _b_	6.55 ± 1.85 _a_	6.76 ± 1.84 _b,c_	5.52 ± 2.46 _b_
Almost all the time	7.49 ± 1.94 _b,c_	7.61 ± 1.79 _b_	7.13 ± 2.09 _b,c_	5.40 ± 2.81 _b_	3.32 ± 2.67 _b_	6.35 ± 2.14 _a_	6.38 ± 2.00 _a,b_	5.52 ± 2.68 _a,b_
**Habitual activity type**								
Low	6.74 ± 1.96 _a_	7.26 ± 1.71 _a_	6.36 ± 2.09 _a_	5.97 ± 2.50 _a_	4.20 ± 2.67 _a_	5.71 ± 2.01 _a_	5.93 ± 1.92 _a_	6.07 ± 2.40 _a_
Medium	7.39 ± 1.65 _b_	7.73 ± 1.55 _b_	7.02 ± 1.81 _b_	5.32 ± 2.53 _b_	3.57 ± 2.74 _b_	6.32 ± 1.89 _b_	6.61 ± 1.79 _b_	5.56 ± 2.45 _b_
High	7.44 ± 1.78 _b_	7.89 ± 1.79 _b_	7.11 ± 2.07 _b_	5.24 ± 2.73 _b_	3.34 ± 2.91 _b_	7.02 ± 1.88 _c_	7.09 ± 1.82 _c_	5.20 ± 2.49 _b_

All values represent Mean (SD). Values in the same column and subtable not sharing the same subscript are significantly different at *p* < 0.05 in the two-sided test of means equality. Habitual activities classification: Activities that do not require physical struggle (reading, watching TV): Low, Relaxing activities (walking, gardening, slow biking) some times per week: Medium, Practice sport or intense physical activities: High.

## Data Availability

All relevant data supporting this research that are not presented either in the manuscript or the supplementary section are in the possession of the MeDiWeB consortium P.I., Dr. Paula Pinto.

## Supplementary Materials

The following supporting information can be downloaded at: https://www.mdpi.com/article/10.3390/nu16081238/s1, Figure S1: BMI and SWB items variation of means (SD) in response to 14-MEDAS groups; Figure S2: Normality test (histograms); Table S1: Additional lifestyle factors according to the 14-MEDAS score groups; Table S2: Description of PCA Factors (top 5) vs 14-MEDAS score for Male and Female participants; Table S3: Normality test (Shapiro-Wilk test).
