# Supplementary material for: Exploring the Relationship between Mediterranean Diet Adherence and Subjective Well-Being among Greek and Cypriot Adults
Source: Nutrients. 2024 Apr 21;16(8):1238. doi: 10.3390/nu16081238 (PMC11054782; doi:10.3390/nu16081238)
Supplement: Supplementary file 1 [file nutrients-16-01238-s001.zip › nutrients-2919361-supplementary.pdf]

Table S1: Additional lifestyle factors according to the 14-MEDAS score groups

	14-MEDAS_Groups					
	Low (<5)		Medium (6-9)		High (>10)	
	N	%	N	%	N	%
Sport frequency						
Never	59	31.1%	96	13.7%	0	0.0%
Occasionally, but not regularly	58	30.5%	228	32.5%	14	31.8%
Regularly, less than 150 minutes per week	38	20.0%	160	22.8%	12	27.3%
Regularly, 150 minutes or more per week	35	18.4%	217	31.0%	18	40.9%
Daily activity						
I normally sit down and I don´t walk very much	104	19.4%	403	75.3%	28	5.2%
I spend some time walking but I don´t make any strenuous effort	61	20.6%	223	75.3%	12	4.1%
I spend quite a lot of time walking and I make frequent strenuous efforts	21	24.4%	62	72.1%	3	3.5%
I make a lot of strenuous efforts, hard work, and activity	3	20.0%	11	73.3%	1	6.7%
Nighttime sleep						
Less than six hours per night	41	21.6%	124	17.7%	4	9.1%
Six to seven hours per night	73	38.4%	340	48.6%	21	47.7%
Seven to eight hours per night	55	28.9%	196	28.0%	16	36.4%
Eight to ten hours per night	20	10.5%	37	5.3%	3	6.8%
More than ten hours per night	1	0.5%	2	0.3%	0	0.0%
Siesta						
No	107	20.5%	392	75.0%	24	4.6%
Yes, occasionally	59	19.8%	228	76.5%	11	3.7%
Yes, frequently	24	20.9%	82	71.3%	9	7.8%

Table S2: Description of PCA Factors (top 5) vs 14-MEDAS score for Male and Female participants

	14-MEDAS_Groups											
	Low (<5)				Medium (6-9)				High (>10)			
	Male		Female		Male		Female		Male		Female	
	Mean ± SD	Median (IQR)	Mean ± SD	Median (IQR)	Mean ± SD	Median (IQR)	Mean ± SD	Median (IQR)	Mean ± SD	Median (IQR)	Mean ± SD	Median (IQR)
Satisfied with life	6.8 ± 2.1	7 (2)	6.9 ± 2.0	8 (3)	7.4 ± 1.6	8 (1)	7.2 ± 1.8	8 (2)	8.4 ± 1.0	8 (1)	8.2 ± 1.2	8 (1)
Life worthwhile	7.2 ± 2.0	7 (1)	7.2 ± 1.7	7 (3)	7.6 ± 1.7	8 (2)	7.6 ± 1.7	8 (2)	8.8 ± 0.6	9 (1)	8.8 ± 0.9	9 (1)
Feeling happy	6.8 ± 2.3	8 (2)	6.4 ± 2.1	7 (2)	7.0 ± 1.9	7 (2)	6.8 ± 2.0	7 (2)	8.5 ± 0.8	8 (1)	7.9 ± 1.4	8 (2)
Feeling worried	4.9 ± 2.8	5 (4)	6.0 ± 2.5	6 (4)	5.1 ± 2.5	5 (4)	5.7 ± 2.6	6 (4)	4.1 ± 3.0	3 (5)	4.2 ± 2.7	4 (4)
Feeling depressed	3.2 ± 2.7	3 (4)	4.4 ± 2.9	5 (5)	3.0 ± 2.6	3 (4)	3.9 ± 2.8	4 (4)	2.0 ± 2.0	2 (3)	2.3 ± 2.2	2 (2)

Figure S1: BMI and SWB items variation of means (SD) in response to 14-MEDAS groups.

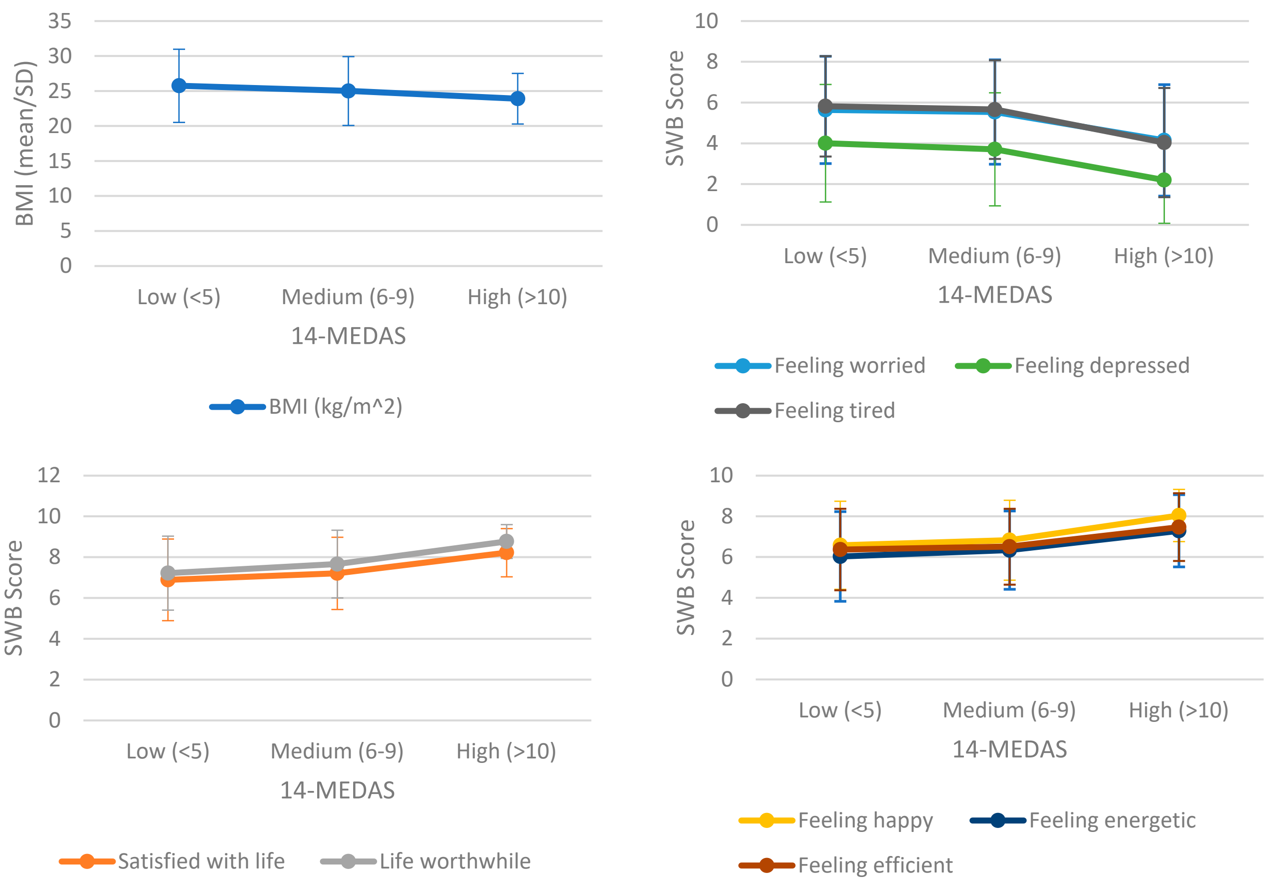

Figure S2: Normality test (histograms).

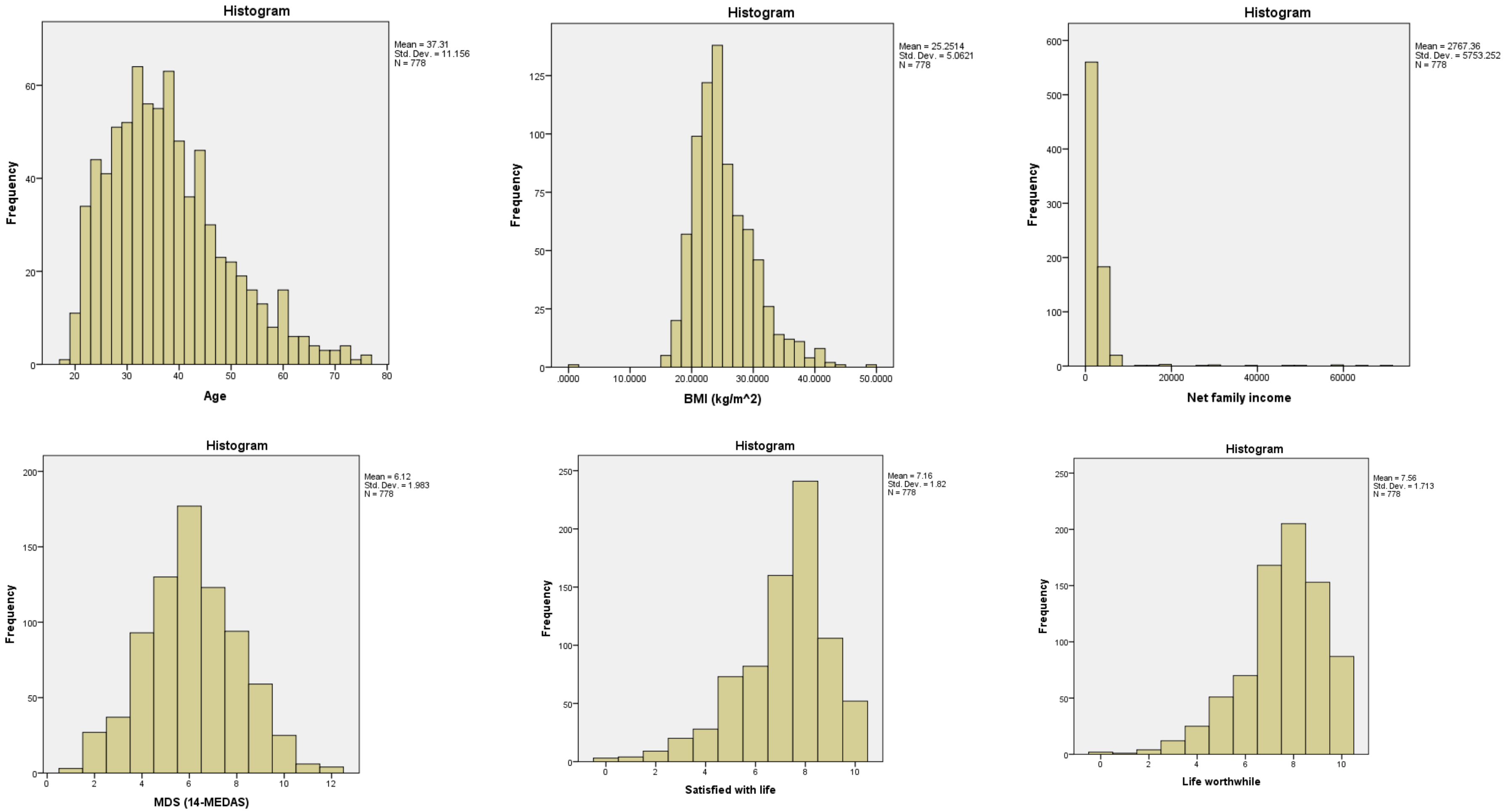

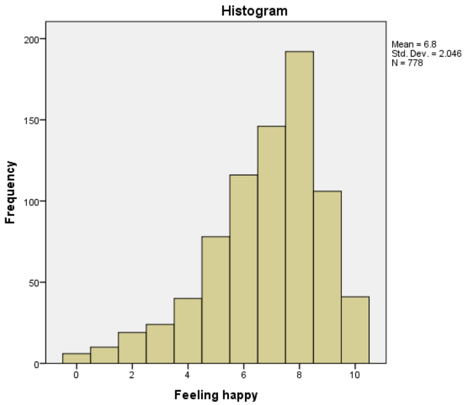
Histogram
Mean = 6.8
Std. Dev. = 2.046
N = 778
Frequency
Feeling happy

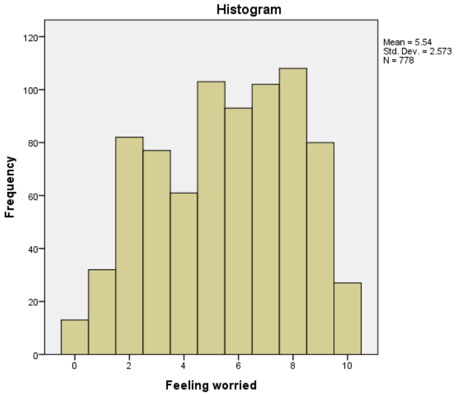
Histogram
Mean = 5.54
Std. Dev. = 2.573
N = 778
Frequency
Feeling worried

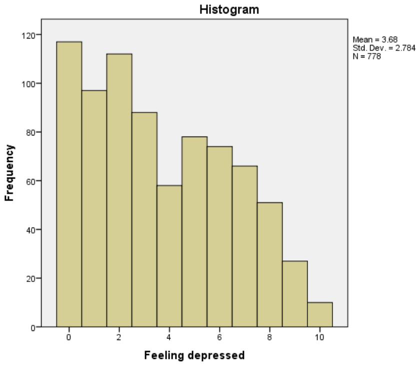
Histogram
Mean = 3.68
Std. Dev. = 2.784
N = 778
Frequency
Feeling depressed

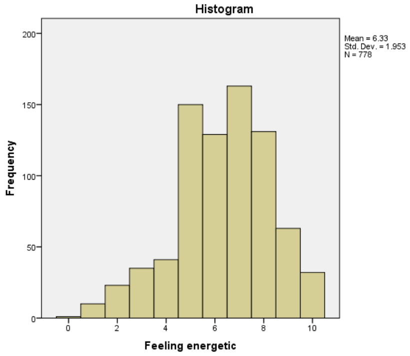
Histogram
Mean = 6.33
Std. Dev. = 1.953
N = 778
Frequency
Feeling energetic

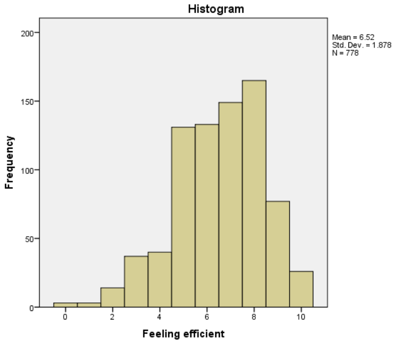
Histogram
Mean = 6.52
Std. Dev. = 1.878
N = 778
Frequency
Feeling efficient

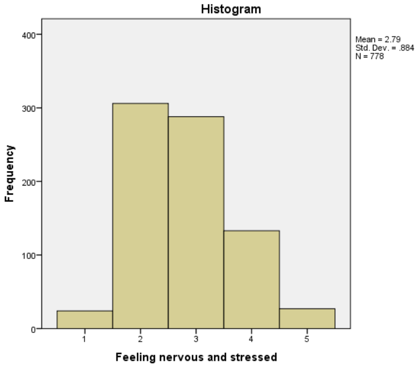
Histogram
Mean = 2.79
Std. Dev. = .884
N = 778
Frequency
Feeling nervous and stressed

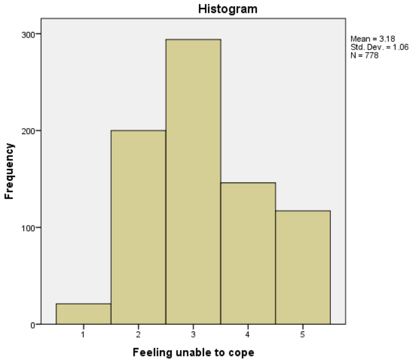
Histogram
Mean = 3.18
Std. Dev. = 1.06
N = 778
Frequency
Feeling unable to cope

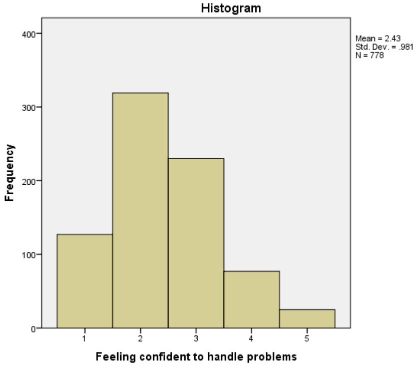
Histogram
Mean = 2.43
Std. Dev. = .981
N = 778
Frequency
Feeling confident to handle problems

Table S3: Normality test (Shapiro-Wilk test).

	Shapiro-Wilk		
	Statistic	df	Sig.
Age	0.955	778	<0.05
BMI (kg/m^2)	0.938	778	<0.05
Net family income	0.281	778	<0.05
MDS (14-MEDAS)	0.976	778	<0.05
Satisfied with life	0.916	778	<0.05
Life worthwhile	0.920	778	<0.05
Feeling happy	0.928	778	<0.05
Feeling worried	0.956	778	<0.05
Feeling depressed	0.932	778	<0.05
Feeling energetic	0.961	778	<0.05
Feeling efficient	0.956	778	<0.05
Feeling tired	0.965	778	<0.05
Feeling nervous and stressed	0.867	778	<0.05
Feeling unable to cope	0.891	778	<0.05
Feeling confident to handle problems	0.887	778	<0.05
